# Dimensions of psychopathology associated with psychotic-like experiences: Findings from the network analysis in a nonclinical sample

**DOI:** 10.1192/j.eurpsy.2023.2429

**Published:** 2023-07-13

**Authors:** Maksymilian Rejek, Błażej Misiak

**Affiliations:** Department of Psychiatry, Wroclaw Medical University, Wrocław, Poland

**Keywords:** early intervention, phenomenology, psychopathology, psychosis, network analysis

## Abstract

**Background:**

Psychotic-like experiences (PLEs) are associated with a variety of psychopathological symptoms. However, it remains unknown which dimensions of psychopathology are most closely related to the occurrence of PLEs. In this study, we aimed to analyze the association of PLEs with various domains of psychopathology.

**Methods:**

A total of 1100 nonclinical adults (aged 18–35 years, 51.4% females) with a negative history of psychiatric treatment were surveyed. Assessment of psychopathology was performed using self-reports. Symptoms associated with PLEs were explored as continuous variables and based on clinically relevant thresholds using two separate network analyses.

**Results:**

In both network analyses, PLEs were directly connected to obsessive-compulsive disorder (OCD) symptoms, manic symptoms, depressive symptoms, and attention-deficit/hyperactivity disorder (ADHD) symptoms. Anxiety symptoms were associated with PLEs only in the network based on threshold scores. Importantly, edge weight for the connection of PLEs and OCD symptoms was significantly higher compared to edge weights of all other direct connections of PLEs with psychopathology in both networks. Edge weight for the connection between PLEs and manic symptoms was significantly higher compared to edge weights for direct connections of PLEs with depressive and ADHD symptoms in the network based on continuous scores of psychopathological symptoms. Edge weights of direct connections of PLEs with depressive, anxiety, and ADHD symptoms did not differ significantly in both networks.

**Conclusions:**

Our findings indicate that PLEs are associated with multiple domains of psychopathology. However, these phenomena are most strongly associated with OCD symptoms regardless of their severity threshold.

## Introduction

Psychotic-like experiences (PLEs) are defined as experiences that are similar to the positive symptoms of psychosis, such as hallucinations, delusions, and disorganized thinking, but do not meet the diagnostic criteria for a psychotic disorder [1]. Notably, PLEs are not uncommon in the general population, with prevalence estimates suggesting that even 7% of individuals may report their occurrence at some point in their lifetime [2]. Moreover, there is evidence that PLEs increase a risk of suicidal behaviors [3]. Although PLEs have traditionally been considered a risk factor for developing psychotic disorders [4–6], a growing body of evidence shows that they may also be indicative of a broader range of mood and anxiety symptoms [7]. Moreover, the longitudinal study by Lindgren et al. [8] revealed that PLEs predict the development of any mental disorder, but not psychosis specifically.

Based on the links between PLEs and nonpsychotic conditions, it has been hypothesized that PLEs might serve as a cooccurring psychopathological phenomenon, which is common in the premorbid stage of various mental disorders [9]. Furthermore, recent research has suggested that they may also represent a unique form of affective dysregulation, distinct from mood or anxiety disorders conceptualized according to known diagnostic systems [6, 10]. While the association between PLEs and affective or anxiety symptoms has extensively been studied, there is limited data on the relationship between PLEs and attention-deficit/hyperactivity disorder (ADHD) or obsessive-compulsive disorder (OCD) symptoms. It has been shown that attenuated psychosis syndrome might be associated with a higher severity of OCD symptoms in college students [11]. Also, there is some evidence that individuals with an established diagnosis of OCD tend to report PLEs that are associated with emotional distress and anxiety traits [12]. Similarly, the study performed in the general population demonstrated that ADHD, together with a number of other psychiatric diagnoses, is associated with the occurrence of PLEs [13]. Interestingly, our group demonstrated that ADHD symptoms are not associated with PLEs in a nonclinical population of young adults after controlling for the effects of other neurodevelopmental risk factors, including childhood trauma history and reading disabilities [14].

The idea that PLEs may serve as an indicator of multi-dimensional affective symptoms has gained interest in recent years, as researchers have begun to explore the complex relationship between these experiences and affective functioning [10]. Despite prior research suggesting associations of PLEs with affective and anxiety symptoms, little is known about their relevance as a multi-dimensional indicator due to the limited scope of previous studies, which have typically focused on only one or two symptom dimensions. For instance, a recent network analysis demonstrated that bizarre experiences are more closely related to manic symptoms, while persecutory ideation might be most strongly associated with depressive and anxiety symptoms [7]. However, the authors of this study did not assess dimensions of psychopathology other than mood and anxiety symptoms.

Taking into consideration, existing research gaps in the field, the present study aimed to disentangle which dimensions of psychopathology are most closely related to reporting PLEs by young adults from a nonclinical population. To address this aim, we adopted a network analysis approach. A network analysis offers a novel conceptualization that affords a more nuanced examination of the interrelatedness and interactions between various psychopathological domains [15]. It provides a more comprehensive and integrated understanding of the pathogenesis and heterogeneity of mental disorders.

## Methods

### Participants

Participants were enrolled by the computer-assisted web interview (March 2023). The information about the study was placed on the online platform used to perform the surveys for research purposes. Participants were enrolled in case of meeting two inclusion criteria, that is, age between 18 and 35 years, and a negative lifetime history of psychiatric treatment. The latter one was recorded using the following question: “Have you ever received any psychiatric treatment?” The snowball sampling methodology was implemented and recruitment procedures were carried out taking into consideration the sociodemographic characteristics of Polish inhabitants reported in 2021. These characteristics are as follows: (1) 51% males; (2) 34% inhabitants aged 18–24 years, and (3) 40% people living in rural areas (cities of up to 100,000 inhabitants: 32%, cities of 100,000–200,000 inhabitants: 9%, cities of 200,000–500,000 inhabitants: 7%, and cities of over 500,000 inhabitants: 12%). Before completing the survey, participants were informed about its confidentiality and anonymous character. The protocol of this study was approved by the Bioethics Committee at Wroclaw Medical University, Wroclaw, Poland (approval number: 99/2023).

### Measures

#### Psychotic-like experiences

To measure PLEs, we used the Prodromal Questionnaire-16 (PQ-16) [16]. The PQ-16 was developed to screen for psychosis risk and includes 16 items measuring the presence of specific PLEs together with associated distress. In the present study, we assessed PLEs in the preceding 4 weeks. We analyzed the subscale measuring the presence of PLEs (true/false responses). Items 1 (“I feel uninterested in the things I used to enjoy”) and 7 (“I get extremely anxious when meeting people for the first time”) were not analyzed in the present study as they might overlap with depressive and anxiety symptoms. The total score ranged between 0 and 14. The Cronbach’s alpha of the PQ-16 was 0.843 in the present study.

#### Depressive symptoms

Depressive symptoms were assessed using the Patient Health Questionnaire-9 (PHQ-9) [17]. The PHQ-9 measures the presence of depressive over the period of preceding 2 weeks. Items are scored on a 4-point scale (responses ranging from 0 – “not at all” to 3 – “nearly every day”). The total PHQ-9 score ranges from 0 to 27. Clinically relevant depressive symptoms were defined as the PHQ-9 score of ≥ 10 [18]. The Cronbach’s alpha of the PHQ-9 was 0.878 in our sample.

#### Manic symptoms

To measure the presence of manic symptoms, we used the Mood Disorder Questionnaire (MDQ) [19, 20]. It includes 13 items measuring the lifetime presence of manic symptoms on a two-point scale (yes or no responses). Two additional questions record the presence of at least two symptoms in the same time period, and associated impairment. Positive scoring for manic symptoms was defined as a history of at least seven manic symptoms, the occurrence of at least two manic symptoms in the same time period, and at least moderate impairment [19, 20]. The Cronbach’s alpha across the MDQ items measuring the lifetime presence of manic symptoms was 0.840 in the present study.

#### OCD symptoms

To measure the presence of OCD symptoms, we used the Obsessional Compulsive Inventory-Revised (OCI-R) [21, 22]. The OCI-R includes 18 items measuring distress associated with OCD and hoarding disorder symptoms over the preceding 1 month. All items are based on a 5-point scale (responses range from 0 – “not at all” to 4 – “extremely”). The total OCI-R score ranges between 0 and 72. To differentiate individuals with OCD and those without a psychiatric diagnosis, the OCI-R threshold score of ≥ 21 has been proposed [21]. The Cronbach’s alpha of the OCI-R was 0.929 in the present study.

#### Anxiety symptoms

The Generalized Anxiety Disorder-7 (GAD-7) was administered to assess anxiety symptoms [23]. It includes seven items measuring the presence of anxiety symptoms in the preceding 2 weeks on a 4-point scale (responses from 0 – “not at all” to 3 – “nearly every day”). The total GAD-7 score ranges between 0 and 21. The GAD-7 threshold score of ≥ 10 has been proposed to screen for GAD [23]. The Cronbach’s alpha of the GAD-7 was 0.925 in the present study.

#### ADHD symptoms

The Adult ADHD Self-Report Scale for DSM-5 (ASRS-5) was used to record the symptoms of ADHD [24]. The ASRS-5 is a screening tool for ADHD that is based on 6 items. Symptoms are measured over the period of preceding 6 months. Responses are scored on a 5-scale (from 0 – “never” to 4 – “very often”). Clinically relevant ADHD symptoms have been defined using the ASRS-5 threshold score of ≥ 14 [24]. The total score ranges between 0 and 24. The Cronbach’s alpha of the ASRS-5 was 0.756 in the present study.

### Data analysis

The network analysis was performed using the R software. Variables included in the network were PLEs (the PQ-16 score), OCD symptoms (the OCI-R score), ADHD symptoms (the ASRS-5 score), depressive symptoms (the PHQ-9 score), manic symptoms (the MDQ score), and anxiety symptoms (the GAD-7 score). Two separate network analyses were carried out. The first one included the symptom scores as continuous variables, while the second one was based on predefined threshold scores for PHQ-9, MDQ, OCI-R, GAD-7, and ASRS-5 (clinically relevant symptoms included as binary variables). The PQ-16 score was included as a continuous variable as we excluded two items that might overlap with depressive and anxiety symptoms. Therefore, it was not possible to use the previously defined threshold score [16]. Additional variables included age, gender, the level of education, occupation, and place of residence as potential covariates. Gender (males vs. females), the level of education (higher vs. other), occupation (unemployed vs. other), and place of residence (urban vs. rural) were included as binary variables. There were no missing data.

As the data included both continuous (the measures of psychopathology) and binary variables (gender, the level of education, occupation, and place of residence), the Mixed Graphical Models were used (the *mgm* package) [25]. To improve the prediction accuracy and interpretability of results, the L1-penalized regression (LASSO) was used [26]. The LASSO reduces the number of estimated parameters to avoid indicating spurious associations by shrinking partial correlation coefficients (i.e., small coefficients are estimated as zero). The penalty parameter selection was performed by the Extended Bayesian Information Criterion (EBIC) according to the tuning parameter λ that controls the level of sparsity [27]. The *λ* parameter was set at 0.5 as proposed previously [26].

The resulting network included the measures of psychopathology and covariates as nodes that are connected with edges. The edge thickness indicates the strength of the association between nodes (thicker nodes indicate stronger associations). Blue edges show positive associations, while red edges show negative associations. The central variables (nodes) were indicated by analyzing the node strength. The node strength is the most commonly used indicator of centrality. It is the sum of all edge weights connected to the node [15, 28, 29]. Moreover, the node predictability was calculated. The predictability of specific node is the proportion of variance explained by nodes directly connected to it. Visualization of the network, node strengths, and predictabilities was performed using the *qgraph* package [30].

The *bootnet* package was used to assess bootstrapped differences in edge weights and strength centrality as well as network accuracy and stability [28]. Bootstrapped differences were analyzed by means of calculating confidence interval (CI) for differences in two values (either edge weights or strength centrality). Results are considered significant if the CI value does not include zero. The case-drop bootstrapping with 1000 iterations was carried out to assess stability of the node strength [28]. Stability of the node strength was visualized, and assessed by calculating the correlation stability coefficient (CS-C). The CS-C should be higher than 0.25. The 95%CI of edge weights was analyzed using the nonparametric bootstrap procedure with 1000 iterations. Greater 95%CI values correspond with lower precision in the estimation of edge weights.

## Results

### Descriptive characteristics of the sample

A total of 1100 participants (51.4% females, aged 27.1 ± 5.1 years) completed the survey (Table 1). The majority of them reported secondary education (50.3%) and were employed full-time (51.3%). Most frequently, participants represented urban place of residence (61.1%).

**Table 1.** Sociodemographic and clinical characteristics of the sample

	Mean ± SD or *n* (%)
Age, years	27.1 ± 5.1
Gender	
Females	565 (51.4)
Males	535 (48.6)
Other	0 (0)
Education	
Primary	61 (5.5)
Vocational	89 (8.1)
Secondary	553 (50.3)
Higher	397 (36.1)
Occupation	
Unemployed	164 (14.9)
Part-time	170 (15.5)
Student	202 (18.4)
Full-time	564 (51.3)
Place of residence	
Rural	428 (38.9)
Urban (up to 100,000 inhabitants)	351 (31.9)
Urban (100,000–200,000 inhabitants)	101 (9.2)
Urban (200,000–500,000 inhabitants)	87 (7.9)
Urban (>500,000 inhabitants)	133 (12.1)
PQ-16	5.3 ± 4.0
GAD-7	7.6 ± 5.5
Positive GAD-7 screening	365 (33.2)
PHQ-9	9.4 ± 6.2
Positive PHQ-9 screening	460 (41.8)
MDQ	5.5 ± 3.7
Positive MDQ screening	182 (16.5)
OCI-R	22.3 ± 14.7
Positive OCI-R screening	395 (35.9)
ASRS	10.1 ± 4.3
Positive ASRS screening	225 (20.5)

Abbreviations: ASRS; the Adult Self-Report Screening Scale; GAD-7, the General Anxiety Disorder-7; MDQ, the Mood Disorder Questionnaire; OCI-R, the Obsessional Compulsive Inventory-Revised; PHQ-9, the Patient Health Questionnaire-9; PQ-16, the Prodromal Questionnaire-16.

### Network structure

**Figure 1.**
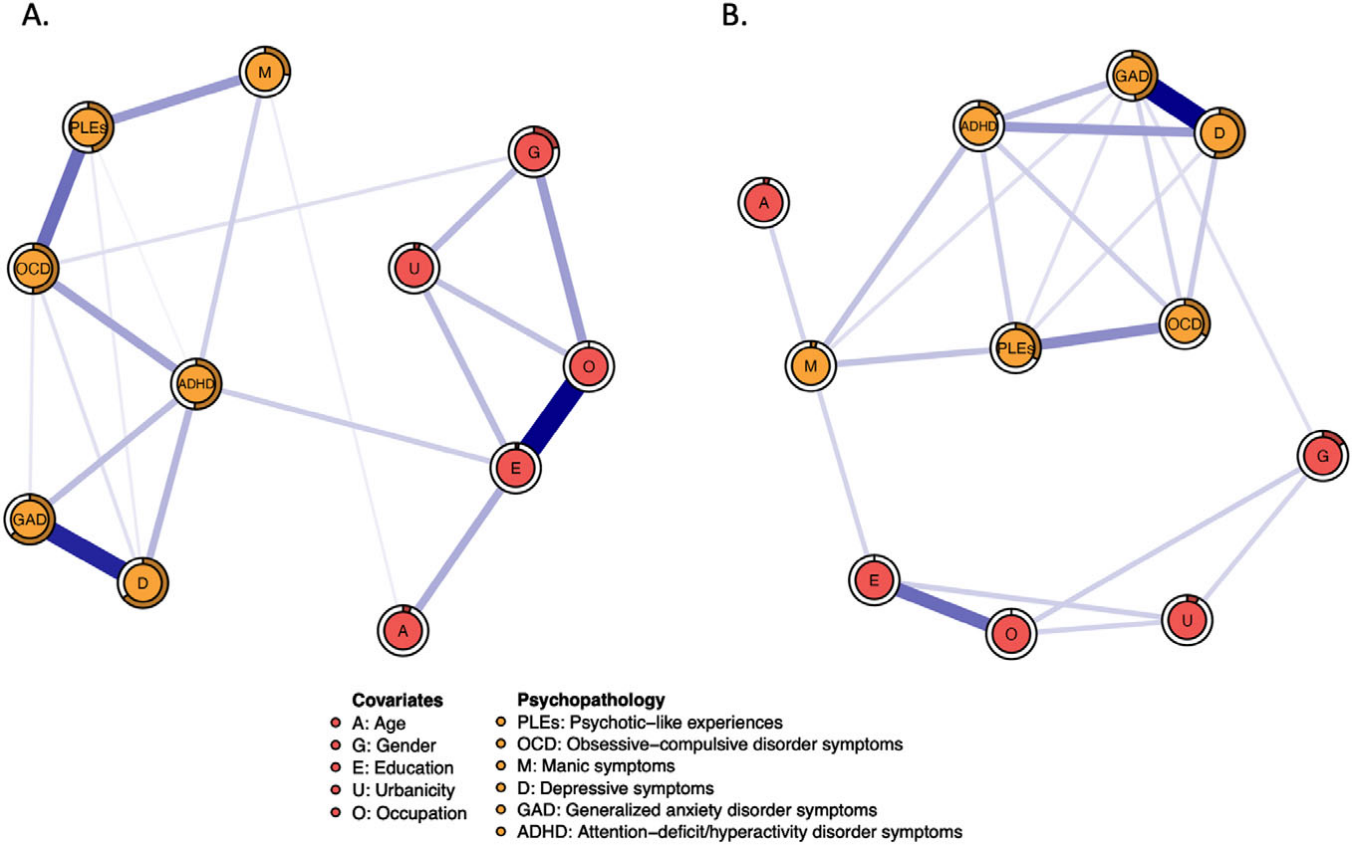
The networks analyzed in the present study based on continuous scores (A) and clinically relevant thresholds (B). The filled part of the rings around nodes represents the predictability of each node (i.e., the proportion of variance of specific node explained by the nodes directly connected to it). Specific variables are shown as nodes that are connected with edges. All edges present positive associations.

The networks analyzed in the present study are shown in Figure 1. Specific groups of nodes were well-connected and no negative edges were found.

**Figure 2.**
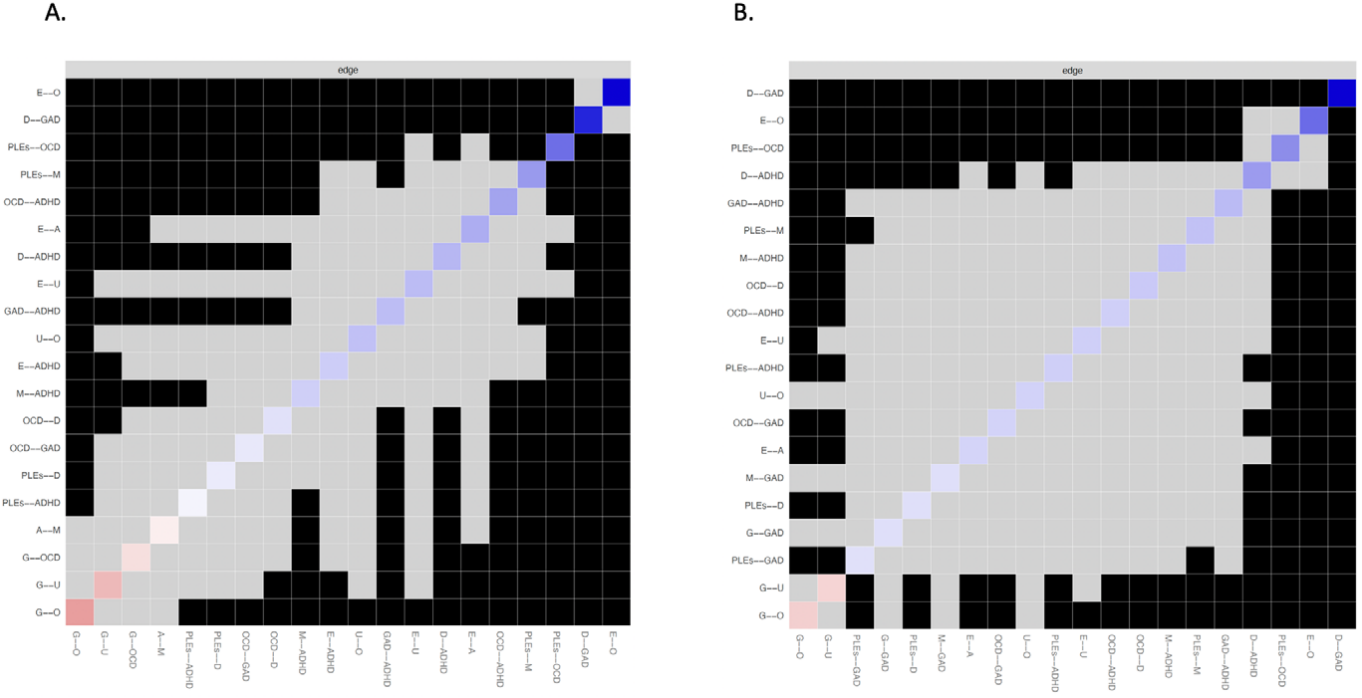
Bootstrapped differences between edge weights in the network analyzing symptoms associated with PLEs based on continuous scores (A) and clinically relevant thresholds (B). Black boxes indicate significant differences. A, age; ADHD, attention-deficit/hyperactivity disorder symptoms; E, education; D, depressive symptoms; G, gender; GAD, generalized anxiety disorder symptoms; M, manic symptoms; O, occupation; PLEs, psychotic-like experiences.

Out of 55 edges, weights of 20 edges (36.4%) were higher than zero in the network analyzing symptoms associated with PLEs based on continuous scores (Supplementary Table S2). The node representing PLEs was directly connected to nodes representing OCD symptoms (weight = 0.392), manic symptoms (weight = 0.275), depressive symptoms (weight = 0.051), and ADHD symptoms (weight = 0.029). Among these connections, the weight of the PLEs – OCD symptoms edge was significantly higher than the weights of other edges (Figure 2A). Also, the weight of the PLEs – manic symptoms edge was significantly higher compared to weights of the PLEs – depressive symptoms and PLEs – ADHD symptoms connections. The weights of connections between PLEs and ADHD symptoms as well as between PLEs and depressive symptoms did not differ significantly. The node of anxiety symptoms was not directly connected to the node of PLEs.

Similarly, the network analysis of clinically relevant symptoms associated with PLEs revealed that 20, out of 55 edges (36.4%), were higher than zero (Supplementary Table S2). In this analysis, the node of PLEs was directly connected to nodes representing OCD symptoms (weight = 0.530), manic symptoms (weight = 0.285), ADHD symptoms (weight = 0.224), depressive symptoms (weight = 0.147), and GAD symptoms (weight = 0.139). The weight of the PLEs – OCD symptoms edge was significantly higher than the weights of other edges connecting PLEs with symptom dimensions (Figure 2A). However, in this analysis, the weight of connection between PLEs and manic symptoms was significantly higher only in comparison with the weight of connection between PLEs and GAD symptoms.

### Central nodes

**Figure 3.**
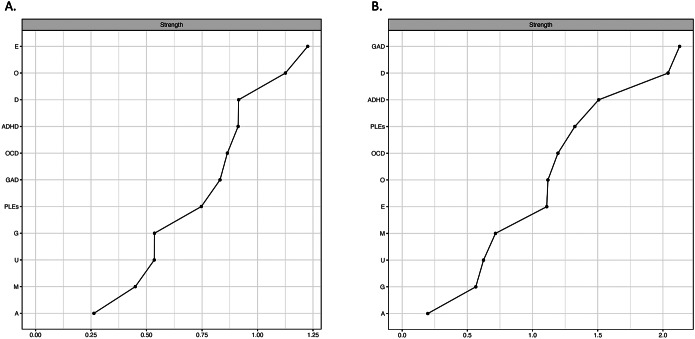
The strength centrality indices in the network analyzing symptoms associated with PLEs based on continuous scores (A) and clinically relevant thresholds (B). A, age; ADHD, attention-deficit/hyperactivity disorder symptoms; E, education; D, depressive symptoms; G, gender; GAD, generalized anxiety disorder symptoms; M, manic symptoms; O, occupation; PLEs, psychotic-like experiences.

**Figure 4.**
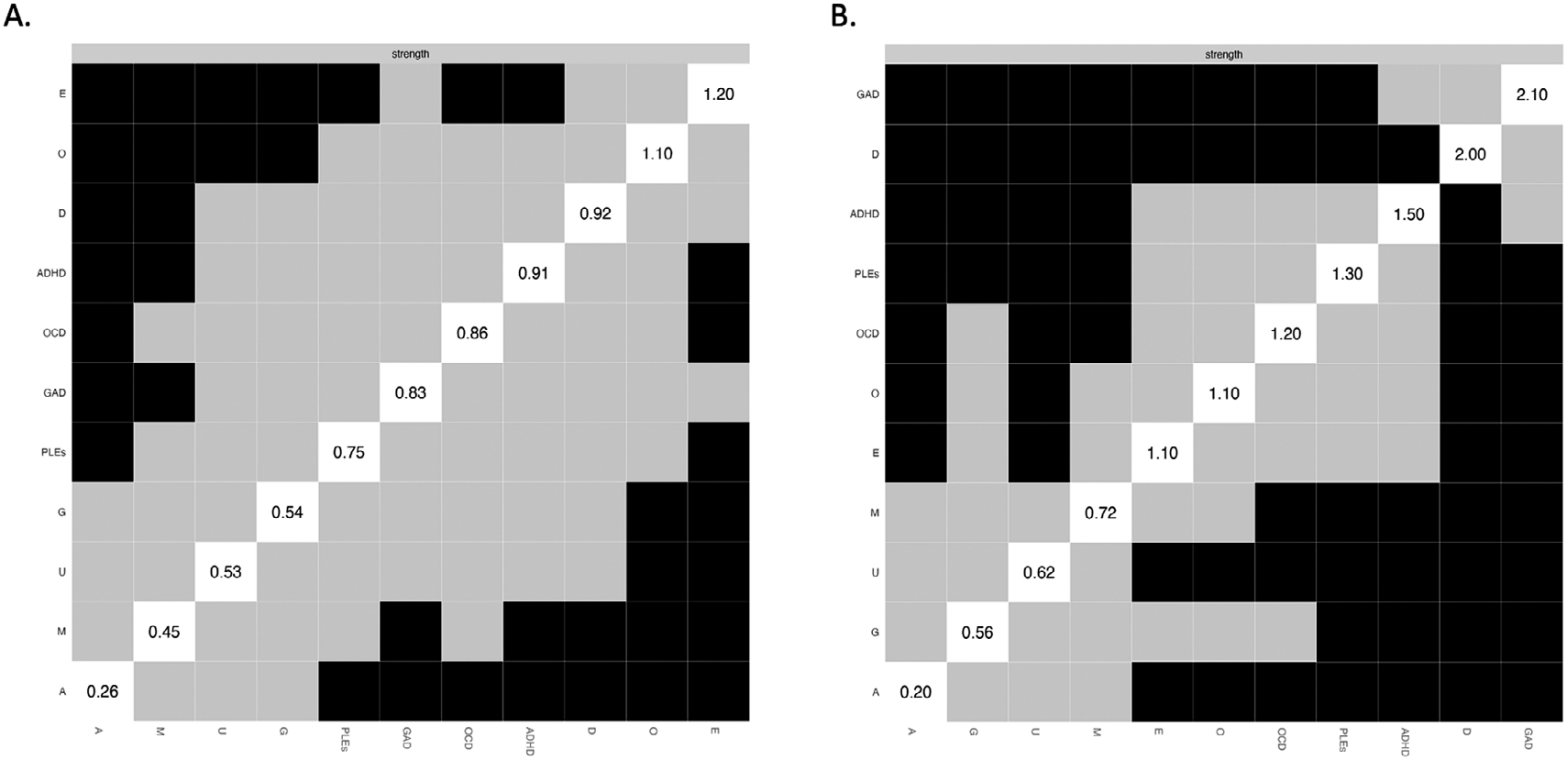
Between-node differences in the strength centrality indices in the network analyzing symptoms associated with PLEs based on continuous scores (A) and clinically relevant thresholds (B). Significant differences are marked with black boxes. A, age; ADHD, attention-deficit/hyperactivity disorder symptoms; E, education; D, depressive symptoms; G, gender; GAD, generalized anxiety disorder symptoms; M, manic symptoms; O, occupation; PLEs, psychotic-like experiences.

The strength centrality indices are shown in Figure 3. The three most central symptom dimensions included depressive symptoms, ADHD symptoms, and OCD symptoms in the analysis of symptoms associated with PLEs included as continuous scores (Figure 3A). However, there were no significant differences in the strength centrality indices between nodes representing specific dimensions of psychopathology (Figure 4A). In turn, in the analysis based on clinically relevant symptoms, the three most central symptom dimensions were GAD symptoms, depressive symptoms, and ADHD symptoms (Figure 4B). The symptoms of GAD had significantly higher strength centrality compared to PLEs and OCD symptoms (Figure 4B).

### Node predictability

The mean predictability across the whole network was 0.306 in the analysis of symptoms associated with PLEs based on continuous scores and 0.195 in the analysis of clinically relevant symptoms associated with PLEs (Supplementary Table S3). This means that the mean-variance of each node explained by nodes directly connected to it was 30.6 and 19.5%, respectively. In the analysis of symptoms associated with PLEs based on continuous scores, the greatest predictability among nodes representing dimensions of psychopathology was obtained for depressive symptoms (0.647), followed by GAD symptoms (0.637), ADHD symptoms (0.512), OCD symptoms (0.503), PLEs (0.469), and manic symptoms (0.271). Similarly, in the analysis of clinically relevant symptoms associated with PLEs the greatest predictability among nodes representing dimensions of psychopathology was obtained for depressive symptoms (0.535), followed by GAD symptoms (0.479), OCD symptoms (0.332), PLEs (0.327), ADHD symptoms (0.156), and manic symptoms (0.038).

### Network accuracy and stability

The node-specific strength was stable when dropping various proportions of data (Supplementary Figure S1). In the analysis of symptoms associated with PLEs based on continuous scores, the CS-C value for node strength and edges was 0.361 and 0.595, respectively. In the analysis of clinically relevant symptoms associated with PLEs, the CS-C was 0.750 (the same value for node strength and edges). These values show that the network models were robust. The bootstrapped 95%CI ranges of edge weights were relatively narrow suggesting sufficient accuracy (Supplementary Figure S2).

## Discussion

This study provides the attempt to employ a network analysis evaluating the intricate association between PLEs and various domains of psychopathology. To our knowledge, this is the first study to implement a network analysis to explore the complex interplay between PLEs and such a wide array of various psychopathological dimensions. We found that all domains of psychopathology, except of anxiety symptoms, are directly associated with the occurrence of PLEs regardless of their operationalization (continuous scores vs. clinically relevant symptoms). Our observations suggest that PLEs may serve as a cooccurring phenomenon shared by multiple domains of psychopathology during a pluripotent period before the manifestation of a specific mental disorder. However, the strongest associations were found for OCD symptoms regardless of their operationalization. The highest predictability value was obtained for depressive symptoms in both networks, indicating that they represent psychopathological symptoms with the highest percentage of variance explained by neighboring nodes in the network. In turn, the highest centrality value was demonstrated for depressive symptoms (the network based on continuous scores of symptoms associated with PLEs) and anxiety symptoms (the network based on clinically relevant symptoms associated with PLEs).

There are important conceptual differences between the node centrality and predictability [31]. Although both parameters are conceptually similar, centrality metrics only allow to order nodes with respect to the number and strength of their direct connections. In turn, predictability provides absolute values showing also what percentage of variance in a given node can be explained by variables that were not included in the network. In this regard, conclusions drawn from the analysis of node predictability might be much more informative for potential interventions. Centrality and predictability metrics are often strongly correlated but the strength of this correlation is lower in case of mixed graphical models that simultaneously analyze binary and continuous variables in comparison with models limited to the analysis of continuous variables [32]. Our study demonstrated that depressive symptoms had the highest percentage of variance explained by neighboring nodes in both networks (over 50%). This observation indicates that interventions focused on targeting depressive symptoms might have the greatest impact on associated domains of psychopathology. Also, in our study, depressive symptoms were directly connected to PLEs. A meta-analysis by Kelleher et al. [33] found that the association between PLEs and depression was moderate, but varied depending on the type and severity of PLEs. Specifically, delusion-like experiences and hallucination-like experiences were more strongly associated with depression than other types of PLEs. Also, the network analysis study of help-seeking adolescents provided some insights into the mechanisms linking PLEs and depressive symptoms [34]. The authors found that both domains of psychopathology might be connected through two pathways, that is, the association between paranoid thinking and distorted body image as well as the association between somatic preoccupation and worry about problems of one’s mind.

Interestingly, we found that OCD had the strongest association with PLEs. It has been shown that the association between OCD and psychotic symptoms might be bidirectional. On one site, there is evidence that people with OCD, especially with early age of onset, are at higher risk of developing prodromal symptoms of psychosis [35]. Also, longitudinal studies have demonstrated that individuals with OCD have a 30-fold higher risk of being diagnosed with schizophrenia [36, 37]. On the other site, individuals at clinical high risk of psychosis are significantly more likely to report OCD symptoms compared to the general population [38]. Finally, on the basis of a meta-analysis, it has been estimated that even 13.6% of individuals with schizophrenia also meet the diagnostic criteria of OCD and about 30% of them report OCD symptoms [39]. Moreover, there is some evidence that individuals with schizophrenia and comorbid OCD diagnosis or symptoms have greater severity of psychotic symptoms [40].

Edge weight for the association between manic symptoms and PLEs was significantly lower than the one for association between PLEs and OCD symptoms. However, this association was significantly stronger than other associations of psychopathological dimensions (i.e., the symptoms of depression, ADHD, and anxiety) with PLEs in the network of symptoms based on continuous scores. It should be noted that the threshold of clinically relevant manic symptoms based on the MDQ requires the occurrence of at least two symptoms in the same time period and their impact on general functioning. In this regard, it needs to be considered that the MDQ scores, especially without the use of generally accepted threshold, might measure other aspects of psychopathology, for example, borderline personality disorder that is highly comorbid with bipolar disorder [41]. Also, psychotic symptoms are highly prevalent in patients with bipolar disorder and those with borderline personality disorder. A recent meta-analysis estimated the pooled lifetime prevalence of psychotic symptoms at 63% in type I bipolar disorder and 22% in type II bipolar disorder [42]. It has also been shown that 20–50% of individuals with borderline personality disorder report psychotic symptoms [43].

As similar to manic symptoms, certain differences with respect to connections between PLEs and anxiety symptoms occurred in both networks. Specifically, PLEs were not directly connected to anxiety symptoms in the network based on continuous scores of psychopathological symptoms. However, in the network of clinically relevant symptoms, anxiety symptoms appeared to be directly connected to PLEs. This difference might suggest that only clinically relevant anxiety symptoms might be associated with the occurrence of PLEs. Of note, causality cannot be established in our study; however, a longitudinal study of children and adolescents demonstrated that persistent high levels of anxiety predict the development of PLEs and psychotic disorders at the age of 24 years [44]. Similarly, cross-sectional studies, based on clinical samples, revealed the association between anxiety disorders and PLEs [45, 46].

Our network analysis also revealed a weak connection between ADHD symptoms and PLEs. These observations are consistent with those obtained by previous studies showing that a diagnosis of ADHD is highly comorbid with other mental disorders [47–49]. It has also been found that a diagnosis of ADHD in childhood might predict the development of psychosis in adulthood [50]. Moreover, a longitudinal study of adolescents revealed that the occurrence of PLEs might predict the development of a wide range of psychiatric disorders, including ADHD, in a 3-year observation [51]. Similarly, it has been shown that PLEs are associated with higher levels of omission and commission errors in divided attention tasks [52].

It is also important to note that the mean predictability across the whole network was higher in case of the analysis of symptoms associated with PLEs as continuous scores compared to the operationalization based on clinically relevant thresholds (30.6 vs. 19.5%). This difference can be explained by the fact that we analyzed the sample of nonclinical adults, who are likely less affected by the occurrence of mental disorders compared to other populations, for example, help-seeking individuals. In this regard, the operationalization of symptoms without threshold scores might better explain their variance in our sample. Nevertheless, it cannot be excluded that a similar study performed in samples with more severe psychopathology, for example, in help-seeking individuals or patients with established diagnosis of mental disorders, would yield different results. However, similar network analyses comparing the correlates of PLEs at various levels of psychopathology have not been performed so far.

Findings from the present study should also be interpreted in light of evidence for shared genetic backgrounds of mental disorders. A recent analysis of data from genome-wide association studies of 25 disorders of the brain demonstrated several correlations of genetic backgrounds for psychiatric disorders that exceeded the possibility of potential diagnostic misclassification [53]. Importantly, there was a lower number of such correlations among neurological disorders. These findings suggest a greater diagnostic validity and knowledge about biological mechanisms in case of neurological disorders. For psychiatric disorders, these findings indicate that their genetic liability crosses traditional diagnostic boundaries. Similar findings were obtained by smaller studies. For instance, a longitudinal cohort and multigenerational family study revealed that OCD is etiologically related to schizophrenia spectrum disorders and bipolar disorder [37]. Another study revealed even broader overlap of genetic backgrounds between mental disorders [54]. The authors of this study found a total of 10 genes to be commonly associated with the risk of OCD, schizophrenia, bipolar disorder, and autism spectrum disorder. Altogether, these findings might suggest that cross-disorder genetic backgrounds may underlie shared correlates within a higher-order structure of psychopathology [55].

There are certain limitations of the present study that need to be considered. It is important to keep in mind that the present study was conducted in a nonclinical population, consisting of young adults without assessment of psychopathology using validated diagnostic instruments. Therefore, caution should be taken when generalizing the findings to clinical populations. Also, various limitations related to the snowball method and sampling accuracy should be taken into consideration [56]. Moreover, the overlap of psychopathological symptoms needs to be considered. For instance, symptoms measured by the ASRS-5 might simply reflect cognitive impairments related to psychosis dimension, mood, or OCD symptoms. In turn, MDQ symptoms might overlap with those related to borderline personality disorder. Another limitation is that the data were obtained through self-report measures, which are subject to the recall bias and may not accurately reflect actual symptoms. Additionally, the cross-sectional nature of the study limits our ability to make causal inferences about the relationship between PLEs and various domains of psychopathology. The sample size of the study was also relatively small, which may limit the generalizability of the findings. Finally, our study did not include other potentially relevant variables, such as personality traits or environmental factors.

In summary, our findings highlight the utility of a network analysis as a method for elucidating complex patterns of connections between various dimensions of psychopathology, including PLEs. The network analysis revealed that PLEs were most strongly associated with OCD, while also showing weaker associations with manic, depressive, and ADHD symptoms. These findings clearly indicate the importance of considering comorbid psychopathology in future research on PLEs. Moreover, our data suggest that PLEs may represent a transdiagnostic marker of psychopathology. Additionally, findings from the present study might hold certain implications for clinical practice indicating that preclinical psychopathology might be associated with the cooccurrence of various symptoms. Among them, PLEs are likely to occur in the context of broader psychopathology covering OCD, mood, anxiety, and ADHD symptoms. However, longitudinal studies based on clinical assessments with valid diagnostic instruments are needed to indicate mental health outcomes most strongly predicted by the development of PLEs.

## Supplementary material

For supplementary material accompanying this paper visit http://doi.org/10.1192/j.eurpsy.2023.2429. click here to view supplementary material
