# Supplementary material for: Dimensions of psychopathology associated with psychotic-like experiences: Findings from the network analysis in a nonclinical sample
Source: Eur Psychiatry. 2023 Jul 13;66(1):e56. doi: 10.1192/j.eurpsy.2023.2429 (PMC10486255; doi:10.1192/j.eurpsy.2023.2429)

**Supplementary Appendix**

**Table 1.** Edge weights in the network analyzing symptoms associated with PLEs based on continuous scores. Abbreviations: A, age; ADHD, attention–deficit/hyperactivity disorder symptoms; E, education; D, depressive symptoms; G, gender; GAD, generalized anxiety disorder symptoms; M, manic symptoms; O, occupation; PLEs, psychotic–like experiences.
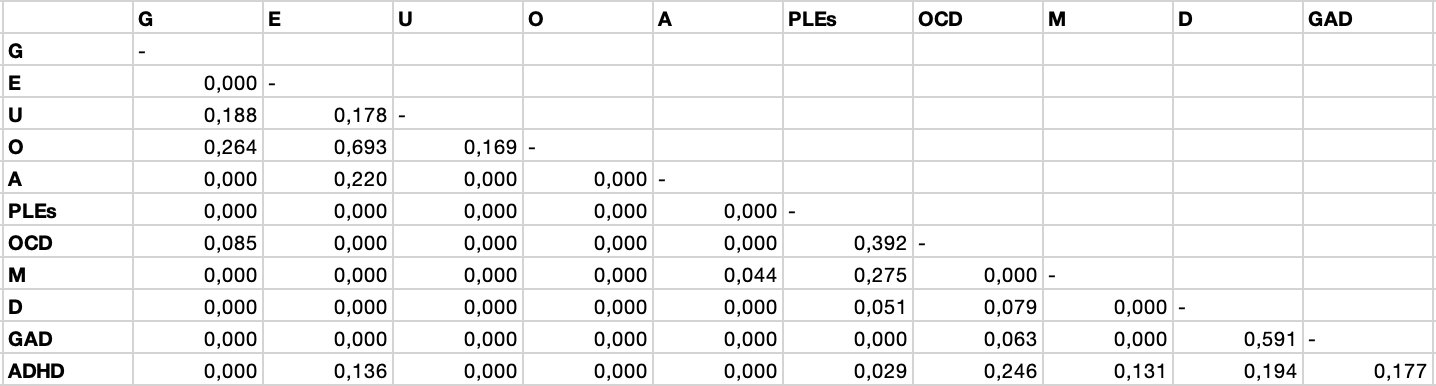


**Table 2.** Edge weights in the network analyzing clinically relevant symptoms associated with PLEs. Abbreviations: A, age; ADHD, attention–deficit/hyperactivity disorder symptoms; E, education; D, depressive symptoms; G, gender; GAD, generalized anxiety disorder symptoms; M, manic symptoms; O, occupation; PLEs, psychotic–like experiences.
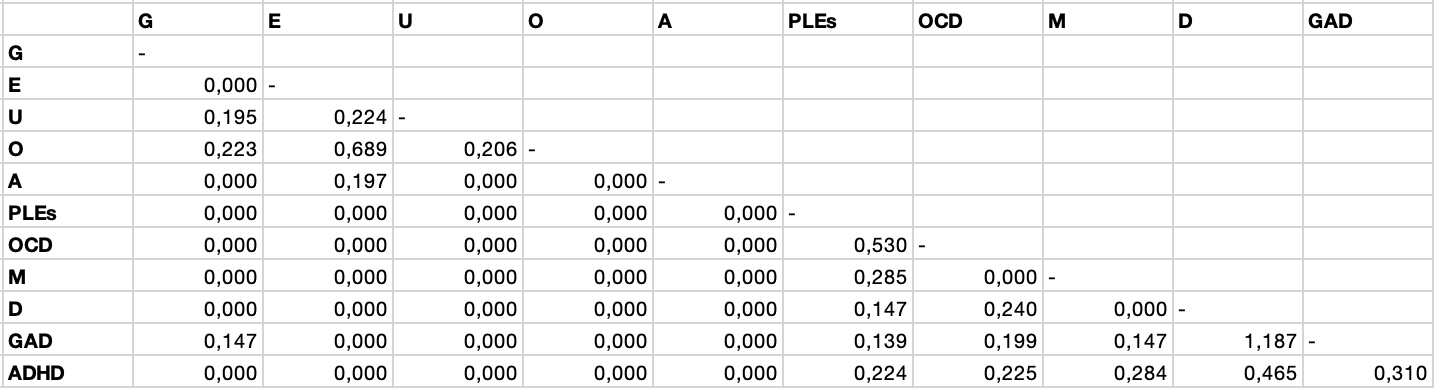


**Table 3.** Node predictability. Abbreviations: A, age; ADHD, attention–deficit/hyperactivity disorder symptoms; E, education; D, depressive symptoms; G, gender; GAD, generalized anxiety disorder symptoms; M, manic symptoms; O, occupation; PLEs, psychotic–like experiences.

| **Node** | **Predictability** | |
| --- | --- | --- |
|  | **Symptoms as continuous variables** | **Clinically relevant symptoms** |
| G | 0.221 | 0.161 |
| A | 0.051 | 0.037 |
| E | 0.020 | 0.000 |
| U | 0.037 | 0.075 |
| O | 0.000 | 0.000 |
| PLEs | 0.469 | 0.327 |
| OCD | 0.503 | 0.332 |
| M | 0.271 | 0.038 |
| D | 0.647 | 0.535 |
| GAD | 0.637 | 0.479 |
| ADHD | 0.512 | 0.156 |

**Figure 1.** Stability of the strength centrality index in the network analyzing symptoms associated with PLEs based on continuous scores (A) and clinically relevant thresholds (B). The red line depicts the strength changes after removing various proportions of data


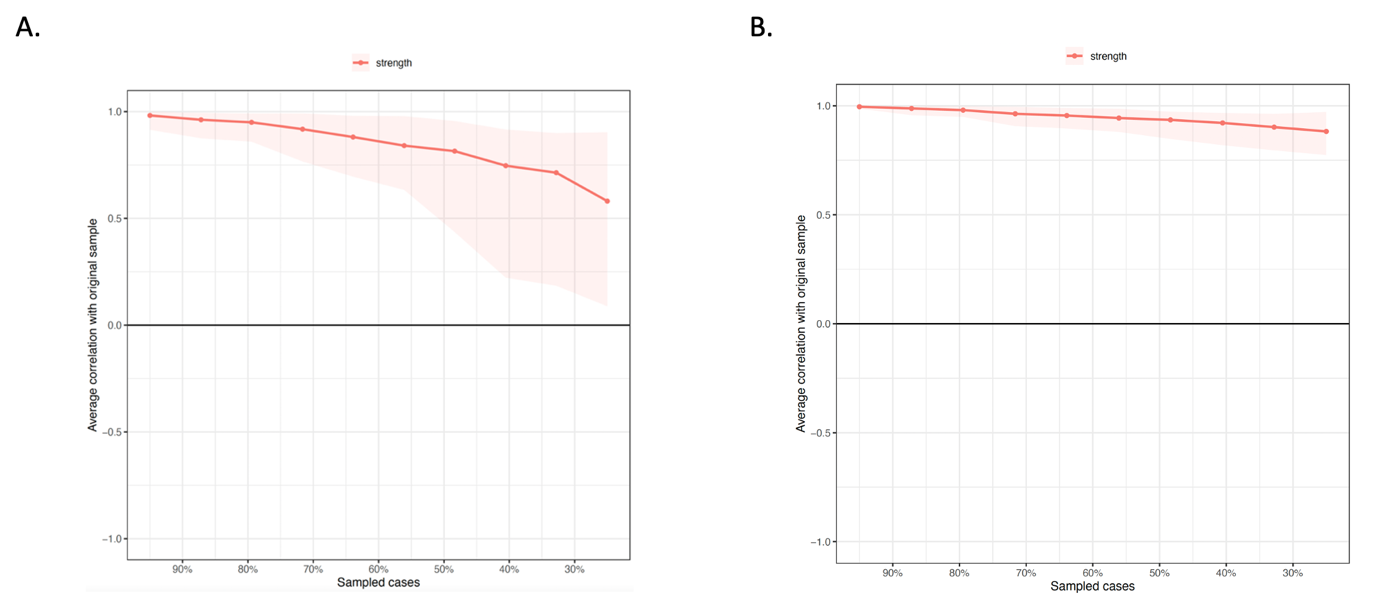


**Figure 2.** Bootstrapped 95% confidence intervals of estimated edge weights in the network analyzing symptoms associated with PLEs based on continuous scores (A) and clinically relevant thresholds (B). The sample values are illustrated with red lines. The bootstrapped 95% confidence intervals are depicted within the grey area. Abbreviations: A, age; ADHD, attention–deficit/hyperactivity disorder symptoms; E, education; D, depressive symptoms; G, gender; GAD, generalized anxiety disorder symptoms; M, manic symptoms; O, occupation; PLEs, psychotic–like experiences.


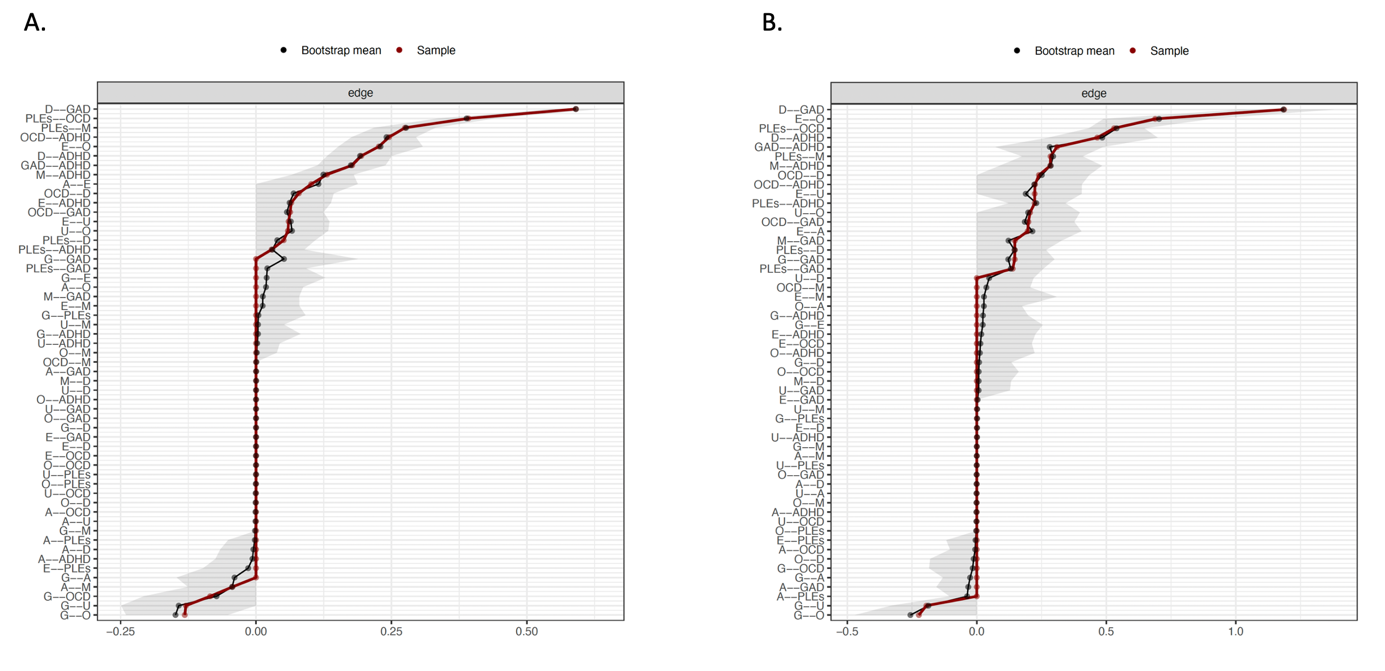

Supplement: Supplementary file 1 [file S092493382302429Xsup001.docx]
